# The social construction of the social epigenome and the larger biological context

**DOI:** 10.1186/s13072-020-00360-w

**Published:** 2020-09-23

**Authors:** Ute Deichmann

**Affiliations:** Jacques Loeb Centre for the History and Philosophy of the Life Sciences, Ben-Gurion University of the Negev, P.O. Box 653, Beer Sheva, 8410500 Israel

**Keywords:** Social epigenetics, Environmental epigenetics, Transgenerational epigenetic inheritance, Cytosine methylation as genomic defense system, Transcription factors, Cellular memory

## Abstract

Epigenetics researchers in developmental, cell, and molecular biology greatly diverge in their understanding and definitions of epigenetics. In contrast, social epigeneticists, e.g., sociologists, scholars of STS, and behavioural scientists, share a focus and definition of epigenetics that is environmentally caused and trans-generationally inherited. This article demonstrates that this emphasis on the environment and on so-called Lamarckian inheritance, in addition to other factors, reflects an interdisciplinary power struggle with genetics, in which epigenetics appears to grant the social sciences a higher epistemic status. Social scientists’ understanding of epigenetics, thus, appears in part to be socially constructed, i.e., the result of extra-scientific factors, such as social processes and the self-interest of the discipline. This article argues that social epigeneticists make far-reaching claims by selecting elements from research labelled epigenetics in biology while ignoring widely confirmed scientific facts in genetics and cell biology, such as the dependence of epigenetic marks on DNA sequence-specific events, or the lack of evidence for the lasting influence of the environment on epigenetic marks or the epigenome. Moreover, they treat as a given crucial questions that are far from resolved, such as what role, if any, DNA methylation plays in the complex biochemical system of regulating gene activity. The article also points out incorrect perceptions and media hypes among biological epigeneticists and calls attention to an apparent bias among scientific journals that prefer papers that promote transgenerational epigenetic inheritance over articles that critique it. The article concludes that while research labelled epigenetics contributes significantly to our knowledge about chromatin and the genome, it does not, as is often claimed, rehabilitate Lamarck or overthrow the fundamental biological principles of gene regulation, which are based on specific regulatory sequences of the genome.

## Introduction

Epigenetics as conceived by Conrad Waddington and David Nanney was originally understood as (i) a “complex of developmental processes” between “genotype and phenotype” in which genes played a major role [1, 2]; (ii) the control mechanisms of gene expression that could lead to different phenotypes of cells with the same genotypes that could be perpetuated during cell division [3]. Research that pursued the questions Waddington raised, was later called developmental genetics, whereas Nanney’s approach was included in research on cellular memory [4]; for the history of epigenetics see [5–8]. The current usage of the term epigenetics in biology is mostly unrelated to Waddington, relating instead to all chromatin and DNA modifications and other transcription regulators that act in the context of chromatin [9].

In the 1970s, heritability was added to the definition of epigenetics, and in 1996 it was suggested that epigenetics is “the study of mitotically and meiotically heritable changes in gene function that cannot be explained by changes in DNA sequences” [10]. With the coupling of DNA methylation and epigenetics, the new epigenetics began to be equated with molecular processes [9]. Meanwhile, research on “histone and DNA-modifying enzymes, nucleosome remodelers, histone chaperones, and chromatin-binding proteins to facilitate transcription factor and polymerase action” [11], was often labelled “epigenetic” and grew rapidly in various scientific fields [8]. According to Tuuli Lappalainen and John Greally, “this transition over time from developmental biology to molecular biology and informatics is substantial, making it unsurprising that there is currently room for major differences of opinion about the most appropriate use of this term.” [12] As a result, epigenetics means very different things to different researchers.

In molecular biology, cell biology and chromatin research, epigenetics can relate to research on chromatin structure and function, DNA methylation and its causes, or the study of the self-perpetuation of signals as a requirement for cells to retain memories of past states. Increasingly, “epigenetics” refers to long non-coding RNA in transcriptional regulation and small interfering RNA as inhibitors of transcription and translation. A major number of epigenetic studies look at the interaction of DNA sequence-specific transcription factors, repressors, and RNA polymerases with histone proteins, chromatin compaction, looping, etc. in gene regulation processes.

Epigenetics has also become an interest in research in ecology, evolutionary biology, and studies of animal behavior as I briefly describe here. Research in these fields does not usually examine epigenetic mechanisms, in most cases relating epigenetics to the inheritance of changes in DNA methylation [13–16]. Unlike in studies in molecular and cell biology, there are no clear-cut definitions of epigenetics. In a study on parental effects and their inheritance in crickets, epigenetic modification as changes in the DNA methylation pattern is only a special case of ‘nongenetic’ inheritance [13]. A study on the behavioral development in sticklebacks holds that a mother’s detrimental condition influences the expression of hundreds of genes in embryos, including noncoding RNAs, genes involved in epigenetic modifications and genes involved in neural growth [14]. Another study on ‘nongenetic’ inheritance in sticklebacks does not suggest mechanisms or mention epigenetics [15]. Many epigenetic studies in organismic biology are speculative about the possible role of epigenetics, for example expressing the hope that “the study of epigenetic inheritance may provide novel insights into previously unexplained aspects of complex ecological interactions” that will lead to a better understanding of adaptation in evolution, or that changes in DNA methylation “may also play a role in conservation” [16].

Most of the biochemical or molecular mechanisms now labelled epigenetic were studied long before the term became popular in the 1990s [8]. Given the multitude of different definitions and interpretations of epigenetics, some researchers consider the current usage of the term highly problematic, unless it is specified. Mark Ptashne, for example, holds that all work on gene regulation, now subsumed under “genetic”, should be termed “epigenetic” instead; he is of the opinion that we should either perceive “epigenetic changes as a subset of gene regulatory changes” or, “in the older Waddington sense”, “we could refer to all developmental gene regulation (including signaling) as epigenetic”; development being a process with no essential changes in DNA sequence [17].

John Greally urges researchers to clarify the way in which they use the term epigenetics and also what should not be labelled epigenetics: “If we mean epi + genetics—the layer of information beyond the genome—it is unclear why we don’t just say ‘transcription regulation’ instead. If our definition is basically a proxy for the mediation of environmental responses, this should not be equated with epigenetic processes at all. My preference is to reserve the term to describe properties involving cell states (cellular reprogramming) and fates […], which are mediated by, but not equivalent to, the many epi + genetic transcription regulators. Whatever your favoured definition, spell it out clearly to let the audience understand what you mean.” [9] (emphasis added).

The above survey shows that though, for historical reasons, molecular and cell biologists in different fields define epigenetics in very different ways, all of them perceive a close relationship between genetics and the mechanisms they study under the label of epigenetics, with borders sometimes blurred. In this article, I do not propose a working definition of epigenetics or the epigenome, rather I use the terms in the way they are understood in the papers that I review here. I use ‘epigenetic marks’ as changes in DNA methylation or histone modifications.

In my opinion, fruitful research is not dependent on a comprehensive definition of a new field of research, as exemplified in the case of molecular biology that lacks an overall definition until today. However, each of its many different subfields has a clear definition and methodology. The same holds true of the concept of the gene, which has remained useful though it has changed drastically over time and is defined very differently in different fields of research. John Greally’s demand that biologists clarify their definition of epigenetics is, therefore, essential for fruitful research.

## The emergence and far-reaching claims of “social epigenetics”

The heterogeneity of definitions and conceptualizations of epigenetics in molecular and cell biology contrasts with the public image of epigenetics that “is more homogeneous than the one described for the scientific community” [18]. A similar homogeneity can be found among social scientists who deal with epigenetics, here called social epigeneticists, in particular sociologists, psychologists, bioethicists, and scholars of public health and of science and technology studies (STS). Social epigenetics or social epigenomics has been acknowledged as a new area of research in various social sciences as well as in the study of organismic biology. The definitions are similar in different areas of research as the following examples show:

A study on animal behavior defines “social epigenomics” as “a new field of research that studies how the social environment shapes the epigenome and how in turn the epigenome modulates behavior” [19]. A sociological study understands “social epigenetics” as research on “how extreme social adversity can lead to later negative health outcomes” with epigenetic mechanisms defined as “molecular modifications that regulate gene activity without changing the DNA sequence” [20]. The authors mainly relate to changes in DNA methylation, overlooking that in this phrasing the definition also applies to conventional gene regulation by DNA sequence specific proteins, but here mainly relates to DNA methylation. A statement from a school of public health describes “social epigenomics” as a field that has emerged in the last few years and looks at “the epigenome as a bridge between social exposure and disease outcomes.” The epigenome here holds “information about all your past exposures—both physical and social—and is shaped by those exposures.” The methodological basis is the relation between poor neighborhood conditions—such as safety concerns, lack of food availability or exposure to environmental toxicants—and DNA methylation in various genes related to stress reactivity and inflammation [21].

The following survey of influential papers by social scientists shows as their common focus transgenerational epigenetic inheritance and the alleged impact of the environment on epigenetic marks or the ‘epigenome’. The authors conceive of epigenetics as the study of molecular mechanisms (not based on DNA sequence) that can turn genes on and off in response to the external environment, with the changed epigenomes (which are not clearly defined but usually relate to the pattern of DNA methylation of a genome) being inherited for many future generations.

The idea of the “social epigenome as a conceptual space,” in which the “inert genome is supplemented by a softer, more adaptable epigenome” that is capable of responding to the environment, is elaborated in [22]. According to the authors, psychologists and sociologists, the ‘epigenome’ describes the overall state of a cell in flux, each point in time yielding multiple cascading possibilities for divergence of individual phenotypes.” The ‘social epigenome’ is defined as the myriad miniscule interactions that are at once socioculturally *and* materially, relationally *and* biologically situated. The authors believe that “with myriad epigenomes, the effects of the wider social and physical environment are translated via biochemical interactions to become an integral part of a fluctuation landscape of gene expression.” They think that “epigenetics has considerable potential to transform social science by embedding mutually regulative reciprocal connections between biological and social processes within the human activities it studies.” They concede, however, that “at present, the absence of consistently established genetic loci and biomarkers for environmental interactions makes it difficult to see a clear pathway for integration of epigenetic markers and social science research.” They demand that “in order for social epigenetics and social science to contribute to the emergence of this putative ‘science of social science’ and to capture meaningful human experience they will both need to change significantly.”

In a widely cited article on epigenetics for the social sciences, sociologist Maurizio Meloni designs a world in which, mediated by epigenetics, boundaries between nature and nurture are blurred (“epigenetics can significantly contribute to undermine established dichotomies between the ‘natural’ and the ‘social’”), with far-reaching consequences for theories of justice [23]. In his opinion, DNA methylation is “the most recognized mechanism of epigenetic mutations,” and he holds that “methylation works as a sort of ‘physical barrier to transcription factors’ and is regulated by nutritional and environmental factors.”

Despite making cautionary remarks about the validity of studies on transgenerational epigenetic inheritance (TEI) in humans, he embraces the notion of epigenetics as environmental TEI and perceives genetics and epigenetics as two entirely different biological processes. He does not question the validity of studies that claim transgenerational effects of chronic disease in individuals prenatally exposed to famine during the Dutch Hongerwinter (Hunger Winter) in 1944–1945 and other studies of the same kind (see “There is no evidence for transgenerational epigenetic inheritance in humans” section). According to him, while 19th century studies showed the “impact of social contexts on the human body,” the new epigenetics studies emphasize “the continuous and plastic interchange of the body with its material surroundings”.

Meloni envisions that the rise of epigenetics will challenge not only the boundary between natural and social inequalities in theories of justice, but also the opposition between biomedical and social constructionist views of the body, with biomedical models focusing on “the endogenous causes of disease,” and social constructionists viewing the “body as the effect of language/power structures.” Against genetic reductionism, he promotes the view of the “openness of the epigenetic body to the world” as “a significant rupture with the mainstream lesson of twentieth century biology, especially genetics.” He perceives “epigenetics, especially when theorized in sophisticated conceptual frameworks,” as a “decisive advance toward what can here be called ‘embodied constructivism’.”

Meloni refers here to the “constructivist interactionism” of Developmental Systems Theory, which invites scholars to “think in terms of non-dichotomous, jointly determined, and reciprocally contingent bio-social factors when explaining human development and social life” and advocates for the equality between the role of DNA and non-DNA elements in explaining development [24, 25]. Meloni’s “embodied constructivism” defines a “non-hierarchical and relational ontology in which social structures can be seen as the sources, as well as the effects, of biological factors.” In his view and that of other social epigeneticists, epigenetics plays an important role for social scientists because it ostensibly limits the power of genetics.

Meloni is exceptional in that he realizes that the idea of the inheritance of acquired characteristics does not necessarily lead to more justice: “ What has not to be forgotten is that the public health implications of soft inheritance are full of problematic and often counter-intuitive aspects.” He cites the Russian geneticist Yuri Filipchenko who in the 1920s expressed the opinion that if Lamarckism was true, “all socially or physically deprived groups, races, and classes of people—such as the proletariat and peasantry and the nonwhite races—would have inherited the debilitating effects of having lived for centuries under deprived conditions.” Interestingly, to preserve the idea of a positive effect of Lamarckism on society, some epigeneticists revoke the notion of epigenetic marks being stable: “Epigeneticists seem in sum more optimistic today about the easy reversibility of epigenetic marks.” [23] Ironically, however, this would eliminate their supposed importance for evolution (see “The stability of the body plan and the genetic, not environmental, origin of novelty in evolution” section).

Also widely cited by social epigeneticists is the “critical introduction to environmental epigenetics for sociologists” by sociologists Hannah Landecker and Aaron Panofsky [26]. They consider environmental epigenetics as a science without taking into account the innumerable objections raised against the idea that the environment can affect the epigenome in a lasting way (see “The environment has no lasting impact on the change of epigenetic marks” section). “Environmental epigenetic research,” they hold, “tracks mechanisms by which social forces—from pollution to nutrition to mothering to traumatic experience—become molecularly embodied, affect gene expression, and induce durable changes in behavior and health.” Mentioning the lack of consensus about mechanisms, they believe that nevertheless, “epigenetic changes meaningful for brains or metabolism can be detected in blood samples or cheek swabs.”

They also point to the efforts of “social epidemiologists tracking what they call the epigenetic signature of depression and posttraumatic stress disorder (PTSD),” and trying to use blood samples to study epigenetic profiles associated with mental disorders. Other suggested practices include a proposed “concept of methylation as a bio-dosimeter for SES [the socio-economic status of a person]: a readout, at the level of DNA methylation, that indexes exposure to social hardship” [26]. The authors do not explain how a blood test could possibly diagnose particular features in view of the fact that all methyl groups are identical. Despite the “lack of certainty about what methylation means and how to measure it in relation to environmental exposures,” they remain optimistic that “environmental epigenetics is a form of knowledge, poised to become a social phenomenon in itself” [26].

A study on the legal and ethical implications of epigenetics, such as the bias against fertile women working in environments “with epigenetic harms,” focuses on long-lasting epigenetic effects induced by nutrition, endocrine-disrupting chemicals, maternal care, maternal stress, and other factors [27]. With alleged transgenerational effects of endocrine-disrupting chemicals, smoking, diet, and alcohol, the authors consider epigenetics to be a “mediator between the environment and the gene.” They also suggest a forecast for evolution, namely that “epigenetic perturbations allow for much more rapid evolutionary change than traditional genetic mutations.” However, as was stated above, their belief in the reversibility of epigenetic changes renders this suggestion highly questionable.

In a frequently cited study on developmental plasticity, psychologist Frances A. Champagne aims at showing that “there is increasing evidence that epigenetic mechanisms, such as DNA methylation, are present across species, are modifiable by the environment, and are involved in developmental plasticity” [28]. She perceives increasing evidence that “variation in the quality of early life experiences can induce epigenetic variation.” Her definition of epigenetic mechanisms as “factors that alter gene expression without altering underlying gene sequence”, as in similar cases, disregards the fact that it includes all DNA sequence-dependent mechanisms of gene regulation. According to Champagne, epigenetic mechanisms appear to play a “significant role in linking the experience of adversity during prenatal and postnatal development to long-term variation in offspring phenotype.” As an example, she presents the experience of abusive caregiving that can have transgenerational effects on DNA methylation of the BDNF (brain derived neurotrophic factor) gene in female offspring in rats.

The incompatibility of genetics and epigenetics is a major focus in an article by bioethicist Dupras et al. reviewing the literature on “epigenetics, ethics, law and society” [29]. The authors define epigenetics as “the study of mitotically and/or meiotically heritable changes in gene function that cannot be explained by changes in DNA sequence.” According to them, “environmental or social epigenetics” became “revolutionary” fields, because they provided the “grounds to revisit some gene-centric theories, long perceived by many as too simplistic and reductionist of human identity, behavior and health.” The cautionary remarks in their review do not address the fact that scientific findings call into question basic foundations of social epigenetics. Instead, the authors remain within the belief system of “environmental” and “social” epigeneticists. One of their criticisms is that the study of the ethical, legal, and social implications (ELSI) of epigenetics was initially highly praised, despite the fact that in-depth analyses of risks and tensions were already arising within the field. They mention, among other things, the storage and sharing of epigenetic data, the risk of stigmatization and discrimination based on individual epigenetic information, and the potential impacts of epigenetics on reproduction and parenting. But they never raise the fundamental question of whether the prevalent epigenetic explanations are scientifically sound.

Their review gives interesting insights into deeper reasons for the creation of social and environmental epigenetics. In their view, social epigenetics was the result of social scientists’ power struggle with genetics: “The genetic era, and its exaggerated emphasis on the biological sources of identity, behavior and health, had created some sort of vacuum for any molecular-scale evidence that would *reinvigorate the epistemic status of social sciences and humanities*.” [29] (Emphasis added) The authors perceive a war against genetics: Social and political theories needed biological support “to be able to counterstrike ongoing geneticization and biomedicalization processes on the same epistemic battlefield.” Therefore, epigenetics, by providing molecular-scale “evidence,” offered a powerful tool to fight the dominance of genetics and increase the epistemic status of the social sciences. Dupras et al. know about the lack of evidence for most of the claims in the field of the ELSI of epigenetics: “Of course, it is crucial to keep in mind that the current epigenetics ELSI literature is mostly anticipatory and speculative.”

Studies labelled epigenetic in psychology, clinical epidemiology, and psychiatry, convey the impression that the notion of trauma being transgenerationally inherited by epigenetic marks has been scientifically confirmed. Thus, though trauma researcher Rachel Yehuda concedes that “there are currently no findings that suggest epigenetic modifications that are specific to PTSD or PTSD risk” [30], she continues to relate the transmission of all kinds of traumata—through terrorism, the holocaust and other factors—to epigenetics, which means DNA methylation patterns. A few years later, she speaks more cautiously about the “putative role of epigenetic mechanisms” in the “intergenerational transmission of trauma effects,” and provides guidelines for the assertion that an effect is truly transgenerational and not only intergenerational [31] (see “There is no evidence for transgenerational epigenetic inheritance in humans” section). According to Yehuda, the “principle of epigenetic plasticity” implies that “changes to the epigenome [changes of DNA methylation] might reset [e.g.] when the environmental insults are no longer present” [31]. If this were the case, the question arises how even intergenerational epigenetic effects could occur.

Clinical psychologist Natan Kellerman even presumes to equate epigenetic marks (“a chemical coating upon their chromosomes”) with numbers tattooed on forearms in Nazi concentration camps. While pointing to the gap between empirical data and his ideas, he envisions “a sort of ‘epigenetic medicine’” for children of trauma survivors [32].

In conclusion, social epigeneticists, applying epigenetics to different contexts, share the following basic convictions and attitudes:

- The belief in genomic plasticity, in which epigenetics opens the genome to environmental cues and interventions;
- The preference for the environment over the gene as the main defining factor for phenotypes and a rejection of what is perceived as genetic determinism;
- The belief in the existence and relevance of transgenerational environmental inheritance in humans through epigenetics, which is often termed ‘Lamarckian inheritance’ despite the fact that most of the examples of supposedly epigenetically inherited traits, such as the effects of trauma or nutrition deficiency, are harmful and not adaptive.

In my opinion, the concept of epigenetics or the epigenome connecting physical and social exposure with human behavior and disease outcomes, and bringing about their inheritance, is mainly socially constructed and not supported by confirmed results of serious research. The idea that scientific knowledge is a social construct was first aimed at the natural sciences, and was brought into philosophical discussions with strong emphasis by the so-called “strong programme in the sociology of knowledge” associated with the work of David Bloor, Barry Barnes, and Harry Collins [33]. It holds that science is mainly a social activity, not privileged to provide access to the understanding of nature as it is. Based on the thesis that any scientific theory is necessarily underdetermined by evidence, social constructionists hold that scientific theories are, to a large extent, the result of extra-scientific factors, such as social processes of ‘negotiation’ and personal interest.

This claim has been well received in science studies like STS because it provides a general and logical reason for the necessity of social and other non-scientific factors in determining the content of scientific theories [34–36]. The denial of a privileged status of (natural) science in its search for true statements about nature was the result of sociology’s power struggle with science. In my opinion, the fact that at least some prominent social epigeneticists promote views that neglect or contradict scientific evidence in what appears to be a disciplinary power struggle with genetics, is an example of social constructivism in the social sciences. Scholars use epigenetics as biological support to limit the power of genetics, or, more forcefully, “to be able to counterstrike ongoing geneticization and biomedicalization processes on the same epistemic battlefield.” [29].

As someone who believes that scientific methodologies enable scientists to generate reliable knowledge and that experimentally corroborated scientific theories largely reflect reality, I consider it irresponsible to promote a scientific or social scientific agenda that is based on unreliable or misleading arguments.

## Scientific evidence that contradicts claims by social and environmental epigenetics; unresolved questions

### It is impossible to separate epigenetics from genetics

In May 2016, an article in the New Yorker about how environmental factors can change the activity of genes without altering the DNA sequence, written by the cancer researcher and author Siddhartha Mukherjee [37], stirred a strong critical response by geneticists, epigeneticists, and other biologists [38]. They criticized that Mukherjee, by emphasizing histone modification and DNA methylation, ignored the primary role of transcription factors and RNA in the transcription process. Mark Ptashne and John Greally pointed to the significance of specificity: “Development requires the highly specific sequential turning on and off of sets of genes. Transcription factors and RNA supply this specificity, but enzymes that impart modifications to histones cannot: every nucleosome (and hence every gene) appears the same to the enzyme.” Not only the specificity of cellular identity but also the response to stress “has been known for decades to be due to the actions of specific DNA binding proteins (and, more rarely, RNA molecules) that regulate gene transcription” [39]. In an exceptional and most commendable response, Mukherjee thanked his critics for their “immensely detailed comments”, admitting to having erred by “omitting key areas of the science” [38].

The exclusion of mechanisms based on DNA sequence as primary cause of gene expression changes is a severe flaw in the arguments of social epigeneticists, too. Emphasizing the supposed role of DNA methylation and histone acetylation in the control of gene regulation, they do not mention transcription factors. As Stephen Henikoff and John Greally commented, transcription factors “actually have many of the required properties of a regulator of cellular memory or a mediator of environmental influences” [4].

Histone modifiers or enzymes that transfer methyl groups to DNA (methyltransferases) lack specific DNA binding domains and are not specifically directed to certain genes. Thus, transcription factors are necessary to target transcriptional regulatory events to specific DNA sequences, sometimes binding to long non-coding RNAs in these events [40]. They also mediate environmental influences on gene activity and maintain cellular memory in a sequence-specific way. In addition, transcription factors are involved in the initial stages of X chromosome inactivation and imprinting, which is then maintained by DNA methylation [12]. Adrian Bird believes that this close interaction between epigenetic marks and genetics is dissolving the distinctiveness of epigenetics. “And I think that’s a good thing” [41].

### The function of DNA methylation is controversial

The role of DNA methylation and histone modification in the biochemical events that regulate genes is still not clearly established and is controversial. There are groups of organisms such as nematodes and certain insects, such as Drosophila, which do not methylate their genomes. It is undisputed that DNA methylation does not silence active promoters of genes, but affects genes that are already silent [42, 43]. It is also generally accepted that one of the major functions of DNA cytosine methylation is its crucial involvement in processes such as transposon silencing, imprinting, and X chromosome inactivation [44–47]. Here, too, DNA sequence-specific factors such as transcription factors or RNAs target the methyltransferases to the respective parts of the genome.

Several authors suggest that the cytosine methylation of repeated DNA sequences and transposons presents a genomic defense system [44, 46]. Timothy Bestor and his co-authors made it clear that despite many correlations between transcriptional activation and demethylation, causation has not been demonstrated, and the available data do not support “the existence of a biochemical system that regulates embryogenesis by programmed methylation and demethylation of regulatory sequences.” They also hold that, “to date there is no reasonable proof of the existence of a complex biochemical system that activates and represses genes via reversible DNA methylation” [46]. The authors criticize the lack of robust criteria in the studies purporting that genes are regulated “by dynamic programmed DNA methylation and demethylation during development.”

Bestor et al. suggest that “mammalian genomic methylation patterns represent an evolutionary adaptation of a genome defense system that endows genomes with the ability to inactivate specific genomic regions in a self-perpetuating manner which is essentially irreversible over the lifespan of the organism.” They agree with Ptashne and Greally [39] that gene activation and repression during development are controlled by well-established and conserved protein-, and RNA-, based mechanisms. Thus, DNA methylation, emphasized most strongly by social epigeneticists as mechanisms of gene regulation, does not appear to play a role in switching genes on and off.

### The environment has no lasting impact on the change of epigenetic marks

According to Adrian Bird, there are no hard data on the influence of the environment on the human “epigenome” [41]. The response to environmental signals is usually mediated by specific proteins such as transcription factors or, in the terminology of Mark Ptashne, recruiters [48].

DNA methylation patterns, once established within a cell by transcription factors, can be replicated and transmitted to daughter cells by the DNMT1 enzyme independently of transcription factors; the same may be true with certain histone marks. This opens the possibility that the methylome may be affected directly by the environment, for example by a severe shortage of enzymatic co-factors such as methyl donors required by the methyltransferases, or by the presence of enzymatic inhibitors such as 2-hydroxyglutarate, which inhibits demethylases. Social epigeneticists may have used this fact for their reasoning, but they have ignored all other facts and contexts, in particular that in almost all cases the environment acts on the phenotype through transcriptional regulation and cellular differentiation. Much of stability and cellular memory is based on gene regulatory networks involving feedback loops [I am grateful to an anonymous reviewer for this information].

According to Edith Heard and Robert Martienssen, epigenetic variation can respond to the environment, but this does not mean that it has any impact on adaptive fitness. Thus, in Drosophila, heat shock or osmotic stress-induced white gene repression can be maternally and paternally inherited for several generations, but then returns to the normal state. In the Agouti mouse, mothers can modulate the coat color of their progeny through a specific diet of methyl donors, but this effect gets lost by the third generation, indicating that the influence of diet is not stable or truly transgenerational [49].

Organisms respond to the environment through the interaction of many factors, most notably specific DNA-binding proteins. In yeast, it was shown that environmental stress such as heat, oxidation, acidity, or starvation, affects various genes in different ways, i.e., the response is DNA sequence specific, with transcription factors and a multiprotein chromatin modifying complex upregulating stress-sensitive genes in response to the stressors [50]. This multiprotein complex is evolutionary conserved; in yeast, it acetylates and deubiquitinates histones [51].

Some studies point to changes of cell fate decisions as a response to the deficiency of micronutrients, or to endocrine disruptors in mice through transcription factors. Endocrine-disrupting chemicals modify the function of the normal endocrine system and represent a major area of interest in epigenetics research [12]. In a well-studied case, mice that were exposed in utero to certain chemicals (of the organotin family, members of which are used as pesticides) accumulated fat from birth to adulthood. These phenotypic effects appeared to be mediated by receptors that cause mesenchymal stem cells to differentiate preferentially into the adipocyte (fat cell) lineage. This means that they do not require the reprogramming of a specific cell type [12].

Testing the hypothesis that victimization of young people across childhood and adolescence is associated with DNA methylation, Marzi et al. showed that such analyses suffered from severe methodological flaws (they were confounded by tobacco smoking and/or did not survive co-twin control tests) [52]. Analyses of six candidate genes in the stress response (NR3C1, FKBP5, BDNF, AVP, CRHR1, SLC6A4) did not reveal predicted associations with DNA methylation. Concluding that their epidemiological analysis of epigenetic effects of early-life stress did not support the hypothesis of robust changes in DNA methylation in victimized young people, the authors recommended that “we need to come to terms with the possibility that epigenetic epidemiology is not yet well matched to experimental, nonhuman models in uncovering the biological embedding of stress” [52]. Heeding this advice would greatly reduce confusion about epigenetic marks.

A recent study showed that, indeed, changes in smoking behaviors were linked to changes in DNA methylation that were dependent on the stimulus across the human genome but independent of genetic and environmental risk factors, as data from twins discordant for smoking behavior did not match [53]. Based on these findings and pointing to methodical problems in social epigenomic studies in general, such as their low statistical power, some sociologists recommend to rely instead on genomic methodology: “With the advent and growing robustness of genomic methodologies, sociologists are in an enviable position to adopt these tools and integrate them into their research” [54].

### There is no evidence for transgenerational epigenetic inheritance in humans

#### Epigenetic inheritance in plants and nematodes

Most scientists reviewed in this article agree about the existence of transgenerational inheritance of acquired traits through RNA in nematodes and through methylation in plants. But proof that transgenerational inheritance has an epigenetic basis in mammals is rare [49].

Transgenerational epigenetic inheritance of unclear function is common in plants. To date, there is no evidence that the inherited traits are adaptive. Epigenetic inheritance in plants is usually associated with transposable elements, viruses, or transgenes and might be, as was suggested for mammals, a byproduct of germline defense strategies [49]. In recent years, a new political movement, which is accompanied by growing sympathy for Stalin, has invoked epigenetics to rehabilitate the flawed experiments on vernalization by agronomist Trofim Lysenko, a protege of Stalin [55]. Vernalization, the influence of temperature and season on the flowering time of plants, was discovered by the German botanist Gustav Gassner in 1918 and then widely applied by Lysenko, who claimed that the effects of vernalization were inherited [56]. The lack of scientific rigour in his work has been analyzed elsewhere, as have the devastating political and economic consequences of Lysenko’s practices (see, e.g., [55, 57].

It has been shown that vernalization that occurs after prolonged periods of cold, results in epigenetic silencing of a floral repressor in a complicated process that involves two protein complexes and methylation. But in contrast to the claims by the new pro-Lysenko movement, the memory of vernalization is not retained in the next generation, because it is robustly reset in the germline and early embryo [49].

Transgenerational epigenetic inheritance has been most reliably demonstrated by many researchers in the nematode *C. elegans*, where small RNAs can enter the germline and mediate heritable transcriptional silencing in subsequent generations (nematodes do not methylate their genomes). An example is the transgenerational inheritance over many generations of small interfering RNAs that target genes that are relevant for the worm’s chemotaxis, nutrition, or virus genome silencing [58–60]. In these studies, Oded Rechavi and his co-authors envisage—but so far are unable to show—that the mechanisms they discovered might provide adaptive advantages for the worm. The mechanisms are gene-based and thus subject to natural selection. These genes, which are “essential for this multigenerational effect” of the transmission of RNAs, target other genes with roles in nutrition [59]. The small RNAs are transcribed and, unlike methyl groups, contain genetic information. For this reason, and because of the hitherto lack of their proven adaptiveness, Rechavi et al.’s statement that “our results, therefore, support the Lamarckian concept of the inheritance of an acquired trait” [60] is not appropriate. Transgenerational inheritance of acquired traits does not have to be Lamarckian, i.e., adaptive and evolutionary meaningful. Results in nematodes cannot easily be applied to humans. Nematodes have a very short generation time and unlike higher animals possess RNA-dependent RNA polymerases that can copy small RNA molecules for many generations. In addition, unlike in *C. elegans*, most of the alleged transgenerationally inherited traits in humans, such as the effects of starvation, are detrimental.

#### The lack of evidence for transgenerational epigenetic inheritance in humans and its rare occurrence in other mammals

Many of the potential examples of epigenetic inheritance that have been proposed for humans, concern inter- rather than transgenerational effects and rarely exclude DNA sequence changes as the underlying cause for heritability [49, 61]. Parental or intergenerational effects occur when the uterus is exposed to toxins, viruses (such as Rubella), detrimental nutritional, or hormonal environments that directly affect the developing embryo and its germline. This exposure usually impacts the first generation, but occasionally also grandchildren. In contrast, transgenerational effects relate to generations that were not exposed to the initial environmental trigger, i.e., to great-grandchildren and beyond.

Intergenerational effects occur in humans and other mammals, but there are two rounds of efficient reprogramming and erasure of DNA methylation in the development of totipotent cells in the early embryo as well as during germ cell differentiation. It is widely believed that this reprogramming prevents the inheritance of most of the epigenetic marks, though some gene loci escape it. Some researchers attribute an evolutionary meaning to it: “Evolution appears to have gone to great lengths to ensure the efficient undoing of any potentially deleterious bookmarking that a parent’s lifetime experience may have imposed,” and they conclude that “although much attention has been drawn to the potential implications of transgenerational inheritance for human health, so far there is little support” [49].

More recently, John Edwards et al. have demonstrated that the dynamics of demethylation and remethylation during early development are more complex than previously assumed [47]. They showed that only sequences that appear to have little evidence of biological function, such as old and inactive transposon remnants, satellite and other repeated DNA, undergo the double wave of demethylation and remethylation. In contrast, other sequences, such as the large majority of CpG island promoters are not subject to these waves of methylation and demethylation because they are unmethylated at all stages. The sex-specific methylation at imprinting control regions is demethylated only in the first round; whereas the small population of young, CpG-rich transposons largely escapes both rounds of demethylation.

The authors showed, moreover, that genomic methylation patterns at regulatory sequences are essentially static during development, and that the demethylation of promoters upon transcriptional activation is likely a consequence rather than a cause of the activation. Citing evidence that only about 10% of the mammalian genome is functional and that among the primary biological functions of DNA methylation are the heritable transcriptional repression of retrotransposons and X chromosome inactivation in female cells, the authors hold that “most DNA methylation is also likely to be without significant biological function” [47].

According to Bernhard Horsthemke, the majority of studies that claim to have demonstrated transgenerational epigenetic inheritance through DNA methylation or sperm RNA—studies that showed responses to environmental metabolic factors (high-fat diet, obesity, diabetes, undernourishment, and trauma) in mice and rats—still await independent confirmation [61]. It is very difficult, Horsthemke says, to provide conclusive proof for transgenerational epigenetic inheritance in mammals, especially humans, because its study is confounded by genetic inheritance, and the impacts of ecology and culture. Some studies, such as those on the transgenerational effects of endocrine disruptors and high-fat diet on the DNA methylome, have been challenged by others.

A key study about the allegedly long-lasting effects of endocrine disruptors reported that the exposure of pregnant female rats to the endocrine disruptor vinclozolin affected male fertility in subsequent generations and that it was associated with epigenetic changes in the germline [62]. Emma Whitelaw drew attention to studies that refuted such claims [63]. A meanwhile widely cited study by Iqbal et al. showed conclusively that these epigenetic changes are corrected by germline reprogramming events in the next generation [64]. According to Whitelaw, the evidence of epigenetic effects lasting for more than one generation as purported in studies on transgenerational effects of the Dutch hunger winter and of PTSD after the world trade center attacks [65, 66] has been inconclusive. She adds the disquieting observation that studies refuting this idea are mainly absent from the literature: “It is very difficult to publish negative results, no matter how important those negative results might be.” As a result, the positive studies “seem to be uncontested to those outside the field” [63].

According to Horsthemke, the increased incidence of cardiovascular and metabolic diseases in the adult offspring of pregnant women who were affected by severe undernourishment during the Dutch “Hongerwinter” was not caused by the transmission of epigenetic information through the maternal germline, but a direct consequence of the exposure in the uterus [61]. He cites studies showing that abnormal DNA methylation patterns can be the result of a mutation in a neighboring gene that affects abnormal promoter methylation in that gene. Since it is dependent on DNA sequence, the transmission of this methylation pattern into the next generation is not an example of transgenerational epigenetic inheritance.

Mayumi Iwasakia, and Jerzy Paszkowskia argue that the prospect of environmental factors, including stress and maternal care, being inherited via epigenetic changes and influencing subsequent generations “are as intriguing as they are troubling, since it is possible to imagine that accumulation of stress memories over several generations could make life decisions difficult” [67]. Investigating the release of detrimental epigenetically suppressed transposons through abiotic stress, they found a mechanism that renders this activation only transient by rapidly resetting stress induced epigenetic states, thus erasing “epigenetic stress memory” and therefore preventing their mitotic propagation and transgenerational inheritance. They showed that this mechanism is conserved between plants and mammals.

#### Methodological problems of epigenome-wide association studies

Epigenome-wide association studies (EWAS), i.e., studies of the changes of DNA methylation in individual genomes or genomes of populations, are widely used to investigate whether DNA methylation changes can be linked to the correlation of disease phenotypes with environmental exposures, in particular those occurring a long time before the phenotype emerged. Statistical problems and the problem of the irreproducibility of such studies are not dealt with here. Instead, this section illuminates the hitherto unresolved problems regarding the interpretation of EWAS, as determined by Tuuli Lappalainen and John Greally [12]. Focusing on the interpretability of even clearly demonstrated DNA methylation changes, the authors reveal multiple problems, including the following:

- “The often vague definitions and terminologies” that are used when discussing epigenetics;
- The fact that DNA methylation can change “in response to a diverse range of influences,” among them the presence of systematic differences in cell-subtype proportions between the groups tested.
- The fact that a large proportion of differences of DNA methylation between individuals can be attributed to DNA sequence. A study by Gertz et al. of a three-generation family and unrelated individuals showed that DNA sequence accounted for up to 80% of the DNA methylation variability [68]. According to the authors, “the majority of variation in DNA methylation can be explained by genotype,” whose influence on patterns of DNA methylation “greatly exceeds the influence of imprinting on genome-wide methylation patterns.” They conclude that the genotype will need to be taken into account when assessing DNA methylation in the context of disease.
- Reverse causation, i.e., the change of DNA methylation as a consequence of transcription, reflecting rather than causing the differences in gene expression. This has been observed in a number of cases (e.g., [69]; and in general, genetic and epigenetic factors are closely interlaced (see “It is impossible to separate epigenetics from genetics” section). Lappalainen and Greally conclude that the fact that “many EWAS do not measure or account for genetic effects on DNA methylation,” is one of the reasons for the current problems of interpretability of EWAS studies [12].

Statistical flaws of studies claiming to have demonstrated transgenerational epigenetic inheritance in humans are indicated by Kevin Mitchell, who points, for example, to noise being interpreted as evidence, or to the justification of sweeping general claims of transgenerational epigenetic effects by tiny statistical differences [70]. Statistical problems are not examined in this article.

## Epigenetics and the broader biological context

### Biological specificity and the constancy of development

Biological specificity—the distinctiveness of individual organisms, species, and higher entities in the hierarchy of taxonomic ranks (genera, orders, classes etc.)—is a fundamental biological principle. Organisms differ, for example, in their body structure and certain molecules, in particular proteins. The differences are now explained by the existence of specific information encoded in the genome that includes specific gene-regulatory processes. Studies that attribute special developmental features to unspecific molecules and events, such as supposedly environmentally triggered DNA methylation and histone modification, disregard the principle of specificity. Likewise, social epigeneticists’ appreciation of plasticity, such as a plastic interaction between genome and environment, disregarding genomic determinism, overlooks the fact that stochasticity and plasticity have long been known to be features of genomic events. Overlooked in particular is the fact that regulatory mechanisms buffer these stochastic events in nature.

Life is characterized by the existence of genome-based regulatory mechanisms that are preserved through evolution by natural selection and that ensure that development leads to a constant outcome despite the widespread stochasticity and plasticity of biochemical reactions. This is demonstrated, for example, in the stability of species over evolutionary times or the similarity of mono-zygotic twins and does not preclude environmental influences. An example is the phenomenon of phenotypic plasticity, the expression of multiple phenotypes from one genome as a widespread adaptation to short-term environmental changes in plants and certain animal species, in particular insects [71]. There are usually only a few different and reversible phenotypes, often two, such as the interchange between asexual and sexual reproduction in certain insects, and not many different ones, which could be expected if the process was stochastic.

### DNA methylation as genomic defense system

There is increasing evidence that one of the main functions of DNA methylation is its contribution to silencing detrimental DNA sequences such as those of transposons, and that in general, methyl groups do not silence genes themselves but are attached to already-inactivated genes. If it can be confirmed that DNA methylation is indeed mainly confined to this silencing role, and if Edwards et al.’s hypothesis that most DNA methylation is “likely to be without significant function” in the mammalian organism [47] will be further corroborated, then the question arises, what conclusions can be drawn from epigenetic wide association studies? So far, there has been no unanimous answer.

### The stability of the body plan and the genetic, not environmental, origin of novelty in evolution

Some social epigeneticists believe that transgenerationally inherited epigenetic marks have played an important role in evolution, and they consider the so-called Lamarckian inheritance rehabilitated. However, the essential reversibility of these marks in response to environmental changes contradicts the fact that the early development of a species always proceeds in the same way, and therefore cannot explain the stability of body plan within species, genus, or higher taxonomic ranks over long periods of time. Without this stability the question of evolutionary change would be meaningless.

Moreover, there is increasing evidence demonstrating that the generation of novelty in evolution is not a response to the environment, such as a new ecological niche, but a result of genetic factors independent of the environment [72, 73]. Novelty can relate to morphological novelty, such as an insect wing or a new body plan, or to genomic novelty (distinguished by sequence similarity or gene architecture). These genetic factors are not, or not primarily, small mutations in the neo-Darwinian sense, but consist, for example, of changes in genomic control regions or co-options of gene regulatory networks to a new developmental address, thereby generating a new morphology [73, 74]. As Doug Erwin has shown, novelties must be generated before they can propagate in newly created ecological niches and become established as evolutionary innovations [73].

The claim that epigenetics rehabilitates the concept of Lamarckian inheritance is not discussed in this article; it has been critically addressed in detail elsewhere [75, 76].

### Science and scientific journals are not immune to fashions and biases

Shortcomings are not confined to epigenetic studies in the social studies of science and behavioural sciences; biases and media hype can also be found in scientific studies of epigenetics. Steven Henikoff and John Greally show how “enthusiasm for epigenetics among researchers” has led, among other things, to a “temptation to refer to any molecules that have been implicated in epigenetic events as epigenetic regulators” [4]. Researchers in different fields label their work as ‘epigenetic’ to increase the likelihood of funding. For this and other reasons, some researchers do not refrain from media hype as has been shown in [77]. A recent example is a newspaper article in which work on the transgenerational inheritance in nematodes is characterized as having rewritten “the basis of genetics” and “upended the Second Law of Biology” (referring to the Weismann barrier) [78].

However, interesting as the new research in nematodes certainly is, it does not affect the basis of genetics or upend a second law of biology. There are no laws in biology comparable to those in physics; exceptions abound to almost every rule. Even if RNA molecules cross the Weismann barrier in nematodes, this would not call into question the general value and basic correctness of the notion of the divergence of germline and soma cells in early development, and a noticeable transgression of the Weismann barrier would lead to fast changes of species properties, but *C. elegans* appears to be a robust species. Media hypes affect the trustworthiness of science.

An apparent bias can also be found among scientific journals including Nature, Cell, and Science that prefer papers that promote transgenerational epigenetic inheritance over articles that critique it. Adrian Bird observed that “the journals tend to love anything that smacks of transgenerational epigenetic inheritance, even if it is only detected in mutant organisms.” Thus, transgenerational inheritance of an epigenetic mark was reported in fission yeast and *C. elegans*, but it occurred only after mutating the enzyme which normally removes this mark [79]. The reported tendencies to hinder the publication of articles that question alleged spectacular results in this regard affect scientific standards and criteria of scientific truth or reliability. In an article in The Guardian, Anne Ferguson-Smith is cited with the warning that transgenerational epigenetic experiments are difficult to perform and can be misinterpreted, but “journals are very excited about this […]. But we must be more cautious” [80]. In the same article, Azim Surani is reported to state that researchers who are not getting positive results are finding their work more difficult to publish, which is feeding hype around the field. Timothy Bestor is quoted as saying that the entire field of transgenerational epigenetic inheritance has been grossly overhyped: “It’s an extremely fashionable topic right now. It’s very easy to get studies on transgenerational epigenetic inheritance published” [80]. He warned that all this excitement has lowered critical standards in science.

## Summary and conclusion

Unlike most epigenetics researchers in developmental, cell, and molecular biology who greatly diverge in their understanding and definitions of epigenetics, social epigeneticists focus mainly on the two topics of alleged environmentally caused and transgenerationally inherited epigenetics. However, most of the reported effects, such as those of trauma and nutritional deficiencies, are in fact results of the direct exposure of the mother, the embryo, and sometimes the embryo’s germ cells to the adverse conditions. DNA methylation does not appear to be involved at all.

The notion of the genome as the most important determining factor for phenotypes means that organisms’ basic characteristics, such as body plans, are generated through the control of their early development by gene networks. It does not mean that every single trait is fully determined by particular genes. The limited effect of the environment on phenotypes was proposed already in the early 20th century by the Danish geneticist Wilhelm Johannsen who equated the genotype with the notion of reaction norm, which referred to the range of potential—reversible—phenotypic variations in different environments, a notion that was developed further by quantitative geneticists. The reaction norm is a characteristic of the genome. Similarly, the phenomenon of phenotypic plasticity, as discussed above, does not call into question the role of the genome as the most prominent determining factor of early development and the constancy of developmental outcomes of most higher animals in different environments.

The emphasis by social epigeneticists on the inheritance of acquired traits and the environment as determining factor for phenotypes is not based on new and reliable scientific results. Rather, at least in some prominent cases, it is the result of a disciplinary power struggle with genetics, in which epigenetics offers to grant the social sciences a higher epistemic status [29]. Since the question of scientific truth or reliability does not seem to matter, I conclude that social epigenetics is to a considerable extent socially constructed to reduce the perceived dominance of genetics. Social epigeneticists select elements from research labelled epigenetics in biology (which were mostly questionable perceptions of epigenetics that could be also be found among some biological epigeneticists), ignoring basic facts in genetics and cellular biology and neglecting contradictory evidence. These views were then extended uncritically to humans. The largest number of misleading, or plainly wrong claims originates from behavioural scientists or psychiatrists regarding the inheritance of trauma.

However, as indicated above, basic scientists are not immune to societal fashions. There is exciting and fascinating inquiry done in chromatin research, the complex events involved in transcriptional regulation, and the silencing of detrimental DNA sequences. But it is undeniable that since this research was labelled ‘epigenetics’, and since the idea of transgenerational inheritance of epigenetic marks has become fashionable, the temptation of epigenetic hype and the danger of lowering critical standards is prevalent, especially in medical and behavioural epigenetics. Adrian Bird commented on the influence of the public opinion on scientific practice: “Because this is something that’s talked about an awful lot, there is the view that the environment influences our epigenome. And I have a skeptical stance on that. Not because I will never believe it no matter what anybody says, but just because I feel there is a great tendency to want it to be true. And I much prefer to see some hard data on that.” [41].

The history of science shows that widespread hype and unfertile approaches were able to affect biological research at the beginning of the 20th century for several decades [81]. But history also shows that unless politics forcibly endows bad science with power, as was the case with Lysenko under Stalin, dead ends and hypes are corrected over time.

Furthermore, history shows that major advances in biology have not only been achieved by replacing concepts and methods in large leaps with better ones, but also by opening up new lines of research, and by integrating previously disparate fields of research. Epigenetics, howsoever defined, is a beautiful example of opening up new lines of research and of synthesis with established fields—genetics and cell biology as well as the known principles of gene regulation—that leading researchers are pursuing today.

## Data Availability

Does not apply.
